# Are estimates of food insecurity among college students accurate? Comparison of assessment protocols

**DOI:** 10.1371/journal.pone.0215161

**Published:** 2019-04-24

**Authors:** Cassandra J. Nikolaus, Brenna Ellison, Sharon M. Nickols-Richardson

**Affiliations:** 1 Department of Food Science and Human Nutrition, University of Illinois at Urbana-Champaign, Urbana, IL, United States of America; 2 Department of Agricultural and Consumer Economics, University of Illinois at Urbana-Champaign, Urbana, IL, United States of America; The University of North Carolina at Chapel Hill, UNITED STATES

## Abstract

A growing body of literature suggests that post-secondary students experience food insecurity (FI) at greater rates than the general population. However, these rates vary dramatically across institutions and studies. FI assessment methods commonly used in studies with college students have not been scrutinized for psychometric properties, and varying protocols may influence resulting FI prevalence estimates. The objective of this study was to assess the performance of standard food security assessment protocols and to evaluate their agreement as well as the relative accuracy of these protocols in identifying student FI. A randomized sample of 4,000 undergraduate students were invited to participate in an online survey (Qualtrics, LLC, Provo, Utah, USA) that evaluated sociodemographic characteristics and FI with the 2-item food sufficiency screener and the 10-item USDA Adult Food Security Survey Module (FSSM; containing the abbreviated 6-item module). Four hundred sixty-two eligible responses were included in the final sample. The psychometric analysis revealed inconsistencies in college student response patterns on the FSSM when compared to national evaluations. Agreement between FI protocols was generally high (>90%) but was lessened when compared with a protocol that incorporated the 2-item screener. The 10-item FSSM with the 2-item screener had the best model fit (McFadden’s R^2^ = 0.15 and Bayesian Information Criterion = -2049.72) and emerged as the tool providing the greatest relative accuracy for identifying students with FI. Though the 10-item FSSM and 2-item screener yields the most accuracy in this sample, it is unknown why students respond to FSSM items differently than the general population. Further qualitative and quantitative evaluations are needed to determine which assessment protocol is the most valid and reliable for use in accurately identifying FI in post-secondary students across the U.S.

## Introduction

A rapidly growing body of literature has developed on the topic of food insecurity (FI), defined as the unavailability of sufficient food, among post-secondary students. Recent reviews estimate that 32.9% to 50.9% of college students in the U.S. experience FI [1,2]. Compared to the 2016 U.S. estimate indicating 12.3% of American households experience FI [3], students seem to be more susceptible to FI. This is concerning, as evidence suggests that FI among adults is associated with lower quality dietary patterns [4–6], more mental health concerns [7,8], diminished physical health [9,10], and greater risk for chronic diseases [11,12]. Studies conducted specifically in college settings indicate that students experiencing FI are more likely to have lower quality dietary patterns, physical health status, and academic success [2]. Though the culmination of findings from studies on university campuses indicate FI is a prevalent issue among college students, the evidence is hindered by limited psychometric testing of food security questionnaires used and thus, related concerns about accuracy in reported estimates.

A variety of assessment procedures have been used to characterize the prevalence of FI among college students. Some studies have used novel questionnaires, such as providing the definitions of food security levels [13,14], evaluating FI on a single item [15,16], using selected items from pre-existing tools [17–21], or making various modifications to standard surveys [22–24]. The primary concern regarding estimates from these reports is that their precision and accuracy in identifying students with FI are unknown. However, the vast majority of studies exploring FI among college students have used the United States Department of Agriculture (USDA) Food Security Survey Modules (FSSM) [25], with several studies utilizing the abbreviated 6-item [15,26–40] and the full individual adult 10-item [41–55] or 18-item household versions [56,57].

Using USDA FSSMs when assessing college students for FI has advantages. The FSSMs were tested extensively when they were originally developed throughout the 1990s, using rhetorical arguments [58,59] as well as qualitative [60] and quantitative evidence [60–63]. Since initial development, the FSSMs have been used widely to evaluate domestic food security prevalence, nationally and locally. This widespread adoption allows for FI estimates to be easily compared across locations, time points, and populations. Therefore, recent studies using the FSSMs when evaluating FI in college students are applying some of the strongest assessment tools available for estimating FI domestically.

Despite the strength of evidence supporting the use of FSSMs, evaluation of their appropriateness for college student populations has not been conducted, as has been the practice for adaptation of other survey instruments to sub-populations [64–67]. There are two critical methodological questions related to FI that warrant evaluation. Broadly, it is worthwhile to know if the FSSMs are appropriate for this audience and if they perform in expected quantitative ways and psychometric patterns. A previous study of students indicated that instrument testing had been performed before use of the 10-item questionnaire; however, results of such testing were unclear, as this evaluation was a small element within a larger non-peer reviewed report with limited description of methods, results, and generalizability [55]. Explicit analyses of the FSSMs against psychometric benchmarks are needed and necessary to support the accuracy and reliability of results. As a second concern, many studies compare their prevalence rates to those published nationally, such as from the Current Population Survey (CPS). Yet, these national procedures use a multi-step FI assessment protocol that includes screening after which many respondents are removed before FI is determined. At present, no prior studies have adopted this multi-step FI assessment protocol when estimating FI prevalence among post-secondary students. Given that the CPS estimates of national FI are the benchmark with which most studies compare their results, it is worthwhile to adopt the multi-step screening methodology and estimate FI prevalence among college students using methods similar to those in the CPS.

The purpose of this study was to address these gaps in the literature related to FI among post-secondary U.S. college students. The objective of the research was to assess the psychometric properties of the FSSM in a student sample and to evaluate the relative accuracy of different protocols (the 6- and 10-item USDA FSSMs, with or without screeners) in identifying high-risk students. It was hypothesized that: 1) the psychometric qualities of the FSSM would be lower than in national samples, and 2) utilizing the screener questions would result in the most relative accuracy when compared to other protocols. These hypotheses were tested in a cross-sectional sample of undergraduate students at a U.S. four-year university by assessment of data fit to the Rasch model and then comparison of each protocol and its performance in predicting students with FI.

## Materials and methods

### Participants

A randomized sample of 1,000 students from each college classification (freshman, sophomore, junior, and senior; 4,000 students total) at one midwestern university was invited from the enrolled student registry to participate in an online survey hosted by the Qualtrics, LLC (Provo, Utah, USA) survey platform. After reviewing a digital informed consent form, respondents consented to participate by selecting “I have read the procedure described above, and I voluntarily agree to participate in this survey.” All study procedures were approved by the institutional review board for research involving human subjects at the University of Illinois at Urbana-Champaign (#16008). Previous studies have reported sociodemographic differences between midwestern college students based on FI status [26], and race/ethnicity results were used as a basis for a chi-squared power analysis (α = 0.05, β = 0.9) using G*Power (Version 3.1.9.2, Heinrich Heine University Düsseldorf). This revealed that a minimum of 134 individuals overall would be needed for bivariate analyses. Given the planned data analyses and projected response rate of online surveys, 1,000 students were sought per college classification to account for incomplete or unusable responses. The survey was administered beginning October 2017 (approximately 7 weeks into the fall 16-week semester) and two reminder emails were sent to eligible respondents, each one full week after the last. Participants who completed the survey were entered into a drawing for one of 250 $10 digital gift cards. To be eligible, students needed to be: 1) between the ages of 18–24 years; 2) enrolled as an undergraduate student at the University of Illinois; and 3) fluent in English.

### Survey

The online questionnaire was designed for completion within approximately 15 minutes on a mobile device or personal computer. Various participant sociodemographic characteristics were collected, including participant’s age, gender, college undergraduate class, race, country of birth, first-generation student status, and living situation. Financial resources were evaluated as a checklist with respondents indicating whether they received monetary support from their family/parent, employment, government/federal grants, scholarships, loans, and/or other sources. For those selected, participants were further asked to estimate the amount they received from each source within a specified timeframe (week, month, semester, or year). An overall estimate of financial support was calculated by converting all values to a 1-semester interval and summing these values. To capture family socioeconomic background, respondents estimated their parent’s income, perceived familial social class, and whether their family used the Supplemental Nutrition Assistance Program and/or free and reduced school lunches while the participant was enrolled in high school.

The surveying procedure was designed to emulate the screening protocol used in the CPS. However, the procedures were adapted for this survey of students. The 2-item screener used by the CPS (Table 1) was included and if a respondent answered ‘No’ and ‘Enough of the kinds of foods that we want to eat’, respectively, they were considered food secure. This screener in the CPS normally references the last twelve months, but a 30-day reference was used to only capture the on-campus experiences of students. In the CPS, high-income households who answer in a secure fashion are not asked the remaining food security questions. In the present study, the screener appeared before the 10-item USDA FSSM, but those who answered affirmatively to these items were not eliminated (i.e., screened out) from answering the FSSM. The impact of the screener was simulated during analyses by following the CPS procedures with additional modifications to the income threshold. Thus, the comparison between differing FI assessment protocols could be tested with a single sample. The screening procedure was first tested using no income estimates and this was followed with a second test where an estimate of ‘financial adequacy’ was developed to approximate an appropriate income threshold for students. To calculate financial adequacy, the overall estimate of financial support per semester was compared with the in-state cost of attendance for an average in-state resident at the University of Illinois [68,69] for the 2017 fall semester. While the survey instrument was designed to capture a variety of sources of financial support, results revealed that students had a tendency to underreport their support level, as only 34.6% of students were considered to have adequate financial support. Given concerns that these financial support estimates and their accuracy were untenable, results from the assessment protocols integrating them with screening procedures are not presented here; however, these results are available upon request.

**Table 1:** Food security questionnaire items and coding of response options as insecure or secure.

Item	Affirmative (Insecure) Response(s)	Negative (Secure) Response(s)
*2-item Food Sufficiency Screener*:		
In the last 30 days, did you ever run short of money and try to make your food or your food money go further?	Yes	No
HH1. Which of these statements best describes the food eaten in your household?	Enough but not always the kinds of food we want to eat, Sometimes not enough to eat, Often not enough to eat	Enough of the kinds of food we want to eat
*10-item Food Security Survey Module*:		
HH2. I worried whether my food would run out before I got money to buy more.	Often true, Sometimes true	Never true, Don’t know
HH3. The food that I bought just didn't last, and I didn't have enough money to get more.*	Often true, Sometimes true	Never true, Don’t know
HH4. I couldn't afford to eat balanced meals.*	Often true, Sometimes true	Never true, Don’t know
AD1. In the last 30 days, did you ever cut the size of your meals or skip meals because there wasn't enough money for food?*	Yes	No, Don’t know
AD1a. In the last 30 days, how many days did this happen?*	≥3 days	1–2 days
AD2. In the last 30 days, did you ever eat less than you felt you should because there wasn't enough money for food?*	Yes	No, Don’t know
AD3. In the last 30 days, were you ever hungry but didn't eat because there wasn't enough money for food?*	Yes	No, Don’t know
AD4. In the last 30 days, did you lose weight because there wasn't enough money for food?	Yes	No, Don’t know
AD5. In the last 30 days, did you ever not eat for a whole day because there wasn't enough money for food?	Yes	No, Don’t know
AD5a. In the last 30 days, how many days did this happen?	≥3 days	1–2 days

Source: Bickel, G., Nord, M., Price, C., Hamilton, W., & Cook, J. (2000). *Guide to measuring household food security*. Retrieved from https://www.fns.usda.gov/guide-measuring-household-food-security-revised

* Items used in 6-item Food Security Survey Module

FI-related questions included the 2-item screener and the 10-item USDA FSSM with a reference period of the last 30 days. The 10-item USDA FSSM queried participants on their experiences with food and financial resources in a series of items that increased in severity. Items of these questionnaires and affirmative responses are included in Table 1. Individuals were experienced FI if they responded affirmatively to three or more items or food secure otherwise. The abbreviated 6-item USDA FSSM is produced from a subset of the 10-item FSSM to reduce participant burden. Therefore, responses of a single sample of participants who answer all items on the 10-item FSSM can be used to simulate response patterns as if only the 6-item FSSM was presented. Items used in the 6-item version are specified in Table 1. Given the reduced number of items, FI is identified if individuals responded affirmatively to two or more items or food secure otherwise.

### Data analyses

Before analyses, survey responses were excluded from the dataset if the respondent was ineligible for the study, the response was a duplicate of a prior response, or less than half of the 10-item USDA FSSM was complete. Once these responses were removed, the averages and distributions of responses on sociodemographic questions were calculated and compared to data (when available) from the entire university undergraduate student body. Descriptive analyses were conducted on all FI assessment protocols to illustrate differences in FI prevalence rates.

The psychometric properties of data were assessed by evaluating whether they fit the single-parameter Rasch measurement model, specifically to identify if items had similar calibrations and item severity order. Per the standardized questionnaire, items on the FSSMs were ordered from least to most severe, and in the general population, the quantity of affirmative responses to each item reflected this order. Similarity to projected response patterns [25] would indicate that the population manages and describes food deprivation similarly and would validate the comparison of rates ascertained among college students with national estimates. For this analysis, item severity parameters, item-infit statistics, and item-outfit statistics were assessed. Item severity parameters, and their ordering, were compared with data collected in the CPS [25]. The estimates of infit and outfit statistics, which reflect the discrimination and consistency of the item responses, respectfully, provided further psychometric indicators of participant response patterns. A conservative range of 0.8 and 1.2 was used to evaluate the fit statistics produced by the 10-item FSSM [70] due to the range of distribution procedures in field settings and the use of FI prevalence studies to directly impact policy. This analysis was not conducted with participants who had missing values on some food security items or ‘extreme’ respondents (those who affirm either all or none of the items), per Rasch model protocol (Appendix C) [25, 71].

The agreement between four potential food security assessment protocols were compared: 1) the 6-item USDA FSSM without a screener; 2) the 10-item USDA FSSM without a screener; 3) the 6-item USDA FSSM with a 2-item screener; and 4) the 10-item USDA FSSM with a 2-item screener. Agreement was calculated for each comparison by taking the difference of the FI designation (yes = 1, no = 0) produced from two protocols. These agreement values were expressed as percentages with 95% confidence intervals based on the standard deviation of the difference.

The relative accuracy of the assessment protocols in identifying participants with FI was assessed by testing each protocol’s designation of the individual (food insecure = 1, food secure = 0) as the dependent variable in logistic regression models and comparing the relative fit of each model. Multivariate logistic regression models were constructed to predict the odds of a student being considered FI based upon risk factors that have been identified as impactful in previous literature [17,20,30,35,41–43,46,51,56]. After testing for potential collinearity, these variables included: race/ethnicity, college classification (Freshman, Sophomore, etc.), social support (living with others), transfer student status (prior enrollment in a community college), first-generation college student status, familial socioeconomic status (comprised of perceived familial social class and use of federal nutrition assistance programs during high school), and sources of financial support [including family, employment, government, scholarship(s), and loan(s)].

To analyze the fit of data to the Rasch model, the ‘ERSRasch’ protocols for SAS 9.4 (SAS Institute Inc, Cary, North Carolina, U.S.) provided by the U.S. Economic Research Service were used. All other statistical analyses were performed in STATA/MP 14.1 (StataCorp, LP, College Station, Texas, U.S.). The complete de-identified dataset as well as the corresponding codebook and statistical analysis files are available as supplementary materials.

## Results

A total of 633 responses were received for the online survey. Of these, 44 respondents did not consent to participate, 22 individuals did not meet inclusion criteria, 80 completed less than half of the 10-item FSSM, and 25 completed the survey twice (the second response or incomplete responses were removed), resulting in a final response rate of 11.5% and sample of 462 participants. Sociodemographic characteristics of participants and comparisons with the institution’s undergraduate student body are reported in Table 2.

**Table 2:** Sociodemographic characteristics of random sample of undergraduate students who participated in an online survey and comparison with university’s undergraduate student body.

Characteristic ^a^	All Participants ^b^ (n = 462)	Undergraduate Student Body ^b^^,^^c^ (n = 33624)
*Age (years)*, mean ± SD	19.6 ± 1.3	20.5 ± NR
*College Classification*, % (n)		
Freshman	27.3% (126)	20.3% (6837)
Sophomore	22.5% (104)	22.9% (7701)
Junior	28.1% (130)	24.7% (8287)
Senior	22.1% (102)	29.9% (10051)
*Race/Ethnicity*, % (n)		
White	51.4% (233)	44.8% (15061)
Black/African American	5.3% (24)	5.9% (1973)
Hispanic or Latino/a	9.7% (44)	11.2% (3748)
Asian/Pacific Islander	27.2% (123)	18.0% (6053)
Other/Mixed	6.4% (29)	20.2% (6789)
*Gender*, % (n)		
Male	35.6% (162)	54.6% (18345)
Female	63.7% (290)	45.4% (15267)
Other	0.7% (3)	0.0% (12)
*Living Situation*, % (n)		NR
Lives alone	10.4% (47)	
Lives with other(s)	89.7% (407)	
*Birth Country*, % (n)		
United States	83.1% (378)	83.4% (28028)
Other country	16.9% (77)	16.6% (5569)
*First-Generation Student*, % (n)	24.3% (107)	20.0% (NR)
*Sources of Financial Support*, ^d^ % (n)		NR
Family	85.1% (382)	
Employment	50.3% (226)	
Government	35.4% (159)	
Scholarship	47.4% (213)	
Loans	38.5% (173)	
Other	1.8% (8)	
*Estimated Parental Income*, % (n)		NR
Under $15000	2.7% (12)	
$15000 to $34999	8.5% (38)	
$35000 to $54999	9.8% (44)	
$55000 to $74999	12.5% (56)	
$75000 to $99999	11.8% (53)	
$100000 to $149999	17.8% (80)	
$150000 or more	18.5% (83)	
Don’t know	18.5% (83)	
*Perceived Familial Social Class*, % (n)		NR
Lower class	9.4% (42)	
Middle class	79.2% (355)	
Upper class	11.4% (51)	
*Familial NSLP use*, % (n)	19.3% (86)	NR
*Familial SNAP use*, % (n)	5.2% (23)	NR

NR = Not Reported, NSLP = National School Lunch Program, SNAP = Supplemental Nutrition Assistance Program

^a^ Missing data: race/ethnicity (n = 9), gender (n = 7), living situation, (n = 8), birth country, (n = 7), first-generation student (n = 22), sources of financial support (n = 13), estimated parental income (n = 13), perceived familial social class (n = 14), familial NSLP use (n = 17), and familial SNAP use (n = 16)

^b^ Sum of column may not add to 100% due to rounding

^c^ Division of Management Information publicly available student enrollment data

^d^ Sum of column will be greater than 100% as participants could select more than one source

Participating students were, on average, aged 19.6 years and roughly balanced across undergraduate college classifications. The majority was born in the U.S., identified as female, and White or Asian/Pacific Islander. Financially, most students received support from their families. The vast majority estimated their familial social class was middle class, though 21% (n = 96) did not estimate their parents’ income. A minority of students indicated limited socioeconomic resources, with a few identifying as families of lower social class or reporting that their families used federal nutrition assistance programs (i.e., free or reduced National School Lunch Program and the Supplemental Nutrition Assistance Program). A minority of respondents indicated that they were first-generation students. In comparison to the entire student body, the recruited sample was younger, less likely to be a college senior, more likely to identify as White or Asian/Pacific Islander and female.

Results when analyzing the 10-item FSSM using the Rasch model are presented in Table 3. This procedure reflects the way that the survey is often used in the field and provides indications of responses on all 10 items. Affirmations of FSSM items ranged from 4.1% on the most severe item, which asked about number of days in which no food was consumed, to 64.5% on the third item that asked if students can afford a balanced meal. Item difficulty estimates reflected these affirmations in quantifiable severity estimates, showing that the final item on the 10-item AFSSM was the most difficult and the balanced meal item was considered the least difficult. The expected response pattern was for items to flow from least to most difficult, and results in this sample deviated from this pattern. The first four items of the survey showed particular deviation, with the ‘balanced meals’ item accruing 5% more affirmative responses than item one, which was expected to be the most commonly affirmed. For infit and outfit statistics, there were deviations outside of the acceptable range of 0.8–1.2 for seven of the ten items evaluated. These non-conforming infit and outfit statistics indicate that there was inconsistency in response patterns and potentially high discriminating value in single items.

**Table 3:** Item response statistics for 10-item adult food security survey module among a random sample of undergraduate students who participated in an online survey (n = 217^a^).

Item	Affirmative responses, n	Affirmative responses, %	Difficulty, estimate (SE)	Item infit, estimate	Item outfit, estimate
Worried run out of food	129	59.4%	5.45 (0.17)	1.22	1.06
Food bought didn’t last	89	41.0%	6.52 (0.18)	1.23	1.23
Cannot afford balanced meal	140	64.5%	5.18 (0.17)	1.30	1.18
Cut or skip meals	125	57.6%	5.56 (0.17)	0.67	0.54
Cut or skip meals, ≥3 days	97	44.7%	6.29 (0.18)	0.63	0.47
Eat less than should	100	46.1%	6.21 (0.18)	0.85	0.78
Hungry, did not eat	79	36.4%	6.80 (0.18)	1.00	0.94
Lost weight	27	12.4%	8.69 (0.24)	1.30	1.46
Did not eat whole day	17	7.8%	9.28 (0.29)	0.95	0.88
Did not eat whole day, ≥3 days	9	4.1%	10.01 (0.37)	0.95	0.63
Mean			7.00		
Standard Deviation			1.62		
Discrimination Parameter			1.00		

Difficulty, item infit, and item outfit are results from a Conditional Maximum Likelihood Rasch model using unweighted data. Fit statistics have an expected ideal value of 1 with a range of 0 to infinity. In this study, the estimates were compared to a preferred range of 0.8 to 1.2. High infit values indicate a weak association of the item to the underlying trait and high outfit estimates indicate inconsistent responses to the item when compared with the overall scale. Low infit and outfit estimates generally reflect high discrimination or Guttman response patterns where the item reflects a rapid transition from mostly affirmative responses to almost none.

^a^ The sample for this analysis only includes responses with complete data (no missing data) and for individuals who affirmed 1 to 9 items on the survey (non-extreme responses)

**Fig 1:**
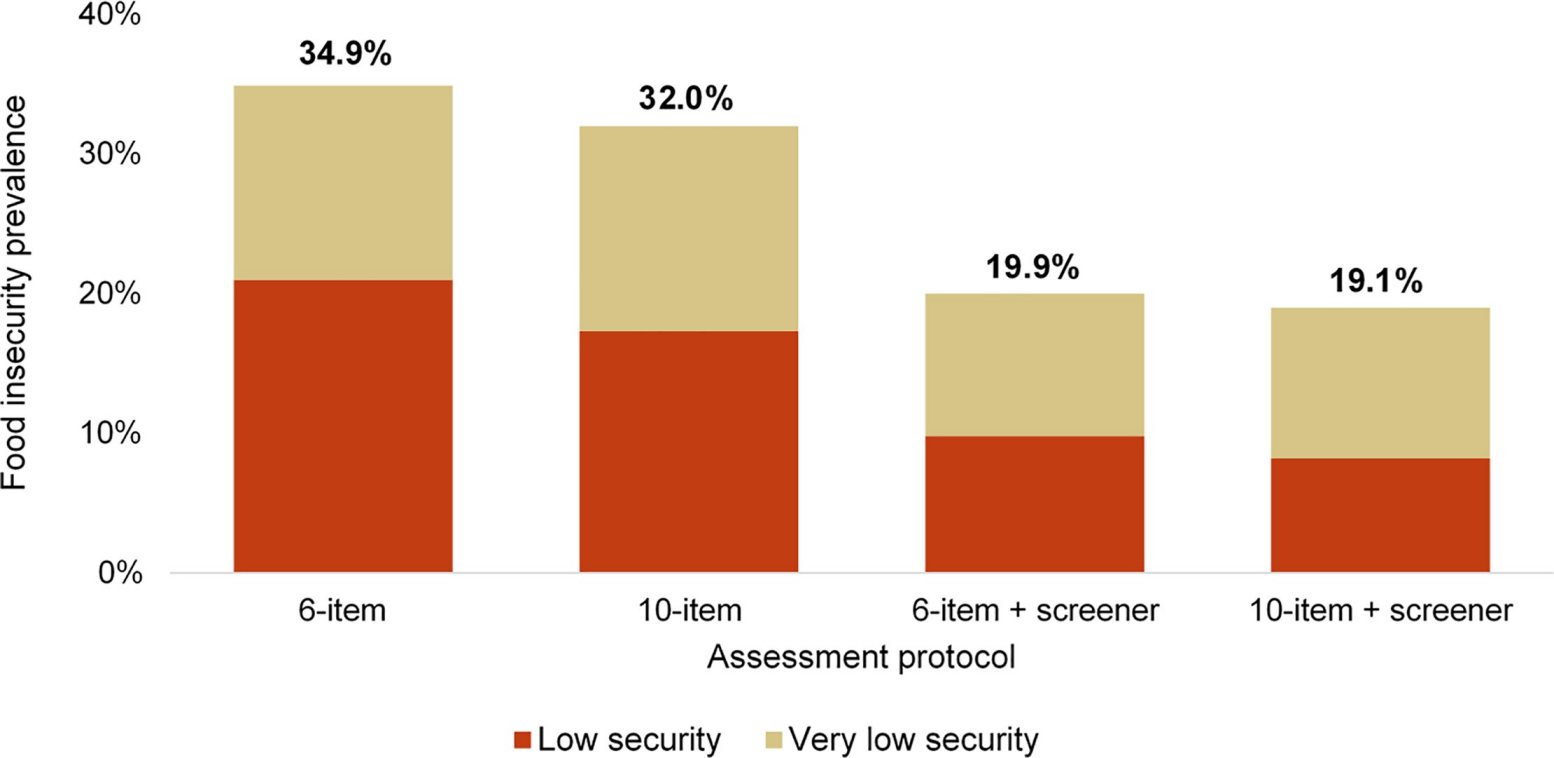
Prevalence rates of food insecurity among undergraduate college students by assessment protocol (n = 462). Note. Assessment procedures and scoring protocols being compared are the: 1) Six-Item Short Form of the USDA Food Security Survey Module; 2) 10-item USDA Adult Food Security Survey Module; 3) Six-Item Short Form with two screening items used in the Current Population Survey; and 4) 10-item USDA Adult Module with two screening items used in the Current Population Survey.

When estimating FI (low and very low levels), Fig 1 reflects how prevalence differs by protocol used. The greatest prevalence of FI was estimated by the 6-item FSSM without screening, and the lowest prevalence was ascertained from the 10-item questionnaire when used with the 2-item screener. Prevalence rates when using the 2-item screener were lower because 327 students (70.8%) answered the screener questions in food secure patterns and thus would not be administered the FSSM under the CPS protocol.

Agreement between FI protocols is displayed in Table 4. Overall, each protocol had strong levels of agreement, but there was variation across protocols. The greatest agreement levels (>95% agreement) were seen when comparing the length of questionnaires (6- or 10-item) with consistent screening protocols. Agreement was diminished when comparing protocols across screening regimes, with percent agreement as low as 84%.

**Table 4:** Agreement of assessment protocols predicting food insecurity (FI) among undergraduate college students (n = 462).

	Protocol			
	6-item	10-item	6-item + screener	10-item + screener
Protocol	% (95% CI)			
6-item	-			
10-item	96.3 (94.2, 97.8)	-		
6-item + screener	85.1 (81.5, 88.2)	86.1(82.7, 89.2)	-	
10-item + screener	84.2 (80.5, 87.4)	87.0 (83.6, 89.9)	99.1 (97.8, 99.8)	-

Agreement for each comparison was based on the difference of the FI designation (yes = 1, no = 0) produced. The mean is expressed as the percentage; the 95% CI is based on the standard deviation.

Multivariate logistic regression models were built for each FI estimation protocol. Due to missing data, regression analyses were run on a sub-sample of respondents (n = 427). The multivariate regression models are presented in Table 5. Sources of financial support had a significant impact on predicting FI status. Across all FI protocols, familial financial support significantly predicted lower odds of experiencing FI, while loans significantly predicted higher odds of experiencing FI. Financial support from employment also increased the odds of experiencing FI in two of the four models. Outside of sources of financial support, a higher college classification (sophomore, junior or senior status) significantly increased the odds of experiencing FI in the majority of models when compared to freshman students. Higher perceived social class also decreased odds for FI, but only in the models predicting FI based on protocols that incorporated the screener. The protocol using the 10-item FSSM and the 2-item screener had the overall best model fit based on McFadden’s R^2^ (0.15) and the Bayesian Information Criterion (-2049.72).

**Table 5:** Comparison of assessment protocols predicting food insecurity (FI) among undergraduate college students based on theoretical predictors in logistic regression models (n = 427).

	Predicting FI with 6-item survey		Predicting FI with 10-item survey		Predicting FI with 6-item survey + screener		Predicting FI with 10-item survey + screener	
Variable	Odds Ratio	Standard Error	Odds Ratio	Standard Error	Odds Ratio	Standard Error	Odds Ratio	Standard Error
Intercept	0.48	0.36	0.64	0.48	0.63	0.54	0.76	0.66
*Race*^a^								
Asian	0.91	0.25	1.06	0.30	0.84	0.29	0.92	0.33
Other	0.88	0.28	0.91	0.29	0.92	0.33	1.05	0.39
*Classification*^b^								
Sophomore	1.83^†^	0.59	1.72	0.58	3.18**	1.39	3.09*	1.42
Junior	2.15*	0.68	2.53**	0.82	4.14**	1.79	4.61**	2.07
Senior	1.37	0.46	1.45	0.51	2.76*	1.24	2.81*	1.31
*Living Situation*^c^								
Live with other	1.27	0.47	1.08	0.40	0.84	0.35	0.77	0.32
*Transfer Student*	1.04	0.40	1.04	0.40	0.77	0.34	0.79	0.36
*First-Generation Student*	1.18	0.34	1.24	0.36	0.97	0.32	0.98	0.34
*Perceived Social Class*^d^								
Middle class	0.71	0.32	0.56	0.26	0.35*	0.17	0.28*	0.14
Upper class	0.80	0.46	0.58	0.34	0.18*	0.12	0.16*	0.11
*NSLP in High School*	1.84^†^	0.67	1.84^†^	0.68	1.40	0.59	1.34	0.58
*SNAP use by Family*	2.22	1.35	1.33	0.78	1.81	1.07	1.32	0.79
*Financial Support*: Family^e^	0.46*	0.15	0.38**	0.12	0.37**	0.13	0.32**	0.11
*Financial Support*: Employment	1.59^†^	0.38	1.65*	0.40	1.46	0.42	1.63^†^	0.48
*Financial Support*: Government	0.85	0.26	0.75	0.23	0.97	0.35	0.96	0.36
*Financial Support*: Scholarship(s)	0.58*	0.15	0.60^†^	0.16	0.43**	0.14	0.40**	0.13
*Financial Support*: Loan(s)	2.19**	0.56	2.20**	0.58	1.65	0.50	1.75^†^	0.54
*Measures of Fit*								
McFadden's R^2^	0.10		0.11		0.13		0.15	
BIC	-1907.53		-1925.39		-2033.40		-2049.72	

NSLP = free or reduced-price National School Lunch Program; SNAP = Supplemental Nutrition Assistance Program; BIC = Bayesian Information Criterion

^†^P<0.10

* P<0.05

** P<0.01

^a^ Relative to White

^b^ Relative to Freshman classification

^c^ Relative to Living Alone

^d^ Relative to Lower Class

^e^ Relative to not having financial support from each source

## Discussion

The aim of this study was to evaluate the standard food security assessment protocols when used among undergraduate college students. Specifically, cross-sectional data from 462 students were used to assess the psychometric properties of the overall survey and then compare the agreement and relative accuracy of four FI estimation procedures. Data supported both proposed hypotheses. First, student responses on the USDA FSSM did not follow the expected difficulty pattern and fit statistics indicated some potential issues on the item-level. Then, when protocols were compared, agreement was generally high. However, agreement was lowest for the FSSMs when paired with the 2-item screener, which resulted in the two lowest FI estimates among students. Finally, this lowest prevalence of FI was supported as the most relatively accurate when predicted by student sociodemographic characteristics.

Results of the Rasch model analysis indicated that the performance of the 10-item FSSM was less than ideal in this college student sample. Affirmative response patterns deviated from the expected flow from least to most severe and the corresponding proportion of affirmative responses. The item that asked students if they could afford a balanced meal was affirmed the most frequently. This keyword ‘balanced meals’ has been scrutinized previously when the FSSMs were evaluated for use in Asian and Pacific Islander populations [64–66]. This item, as well as others, may have keywords that elicit different cognitive responses than expected, based on the formative work used to construct the questions that ultimately resulted in the USDA FSSMs [60,72].

Beyond the deviated order of item difficulty, the Rasch model analysis also revealed that item fit statistics were outside of customary ranges for several items. These statistics reflect ‘noisy’ response patterns, which can result as a product of careless responses due to survey fatigue or limited discriminating power of the questions. The potential fatigue of respondents in this study is less likely given that the FSSM items were placed at the beginning of the online survey, which in and of itself was structured to take less than 15 minutes. Alternatively, limited item discrimination power may be a result of heterogenous responses that participants had to the financial qualifying aspect of each item. When a general adult audience responds to the questionnaire, many would equate an inability to ‘afford’ or have ‘enough money’ for food with the presence and/or adequacy of regular paychecks resulting from employment. In contrast, post-secondary students have a variety of financial situations. An increasing number of college students are employed while enrolled in school [73], but this is one source of support within a larger assortment of resources, both formal or informal, that students may have access to and utilize to ascertain food. The need to consider and calculate these various food and financial support sources while answering seemingly straightforward questions may result in participants ‘satisficing’ and providing a less accurate but convenient response [74]. Given the heterogeneity of support situations, this may explain the erratic response patterns on the survey. However, qualitative studies utilizing cognitive interviewing techniques would be needed to accurately illustrate how students process the FSSMs and to support modifications to the FSSMs for this population.

Until adapted FSSMs are proposed and tested, researchers in the field will need to consider which current FI assessment protocol is appropriate for their sub-population. Findings from the current study comparing protocols provide clear indications that different methods impact FI prevalence estimates, with estimates ranging from 19.1% to 34.9% in a single sample. The protocols tested are a subset of those used previously in the field, and the various agreement levels between methods may, in part, explain the wide range of prevalence estimates previously reported across studies [1,2]. When the 2-item screener was implemented, FI prevalence rates in the sample were reduced by approximately one-third.

Using the 10-item FSSM with the 2-item screener had the greatest ability to identify FI based on established predictor variables. Though all models had limited predictive power, this protocol may provide the most relative accuracy when estimating FI prevalence as well as evaluating the impact of interventions taking place across campuses nationally [57,75]. Furthermore, this protocol more closely resembles those used in the CPS and will allow for more straightforward comparisons of rates of FI among students with those at the national level. However, efforts to simulate the CPS screening that included financial adequacy estimates were unsuccessful in this study. Issues with measurement of financial support in this sample are indicated by the estimate that only 34.6% were considered to have adequate financial support.

Evaluating financial resources among students is inherently complex and is reflected in the methods used to evaluate financial support in the current study. Though efforts were made to create a comprehensive assessment of ‘income’ among students, the estimate showed low predictive power of FI and was ultimately not presented in the current article. Though FI is not synonymous with low-income status, and other factors have mitigating impacts on FI risk [76,77], there is commonly a strong relationship in the general population. Parental income could be used as a proxy for student income, as it is used to calculate the Expected Family Contribution when calculating financial aid offers [78], but this has issues in practice. Specifically, there is the concern that parental income and calculated expected contributions do not resemble actual contributions to students. More practically, many students in the current study selected ‘don’t know’ when asked about their parent’s income, making it difficult to accurately assess. In contrast, asking about the presence of familial financial support may be a valuable proxy as it was a significant protector against FI among students in this population and respondents could more readily provide responses to the discrete question.

The current study provides indications for relative accuracy of differing FI assessment protocols among college students. At its core, accuracy attempts to evaluate whether tests result in an unbiased correct answer. However, it is difficult to evaluate whether a FI assessment protocol is identifying “true” FI, as this is an inherently subjective social condition. In other populations, these accuracy analyses have been conducted by evaluating how different protocols correlated with indicators of food insufficiency, such as use of a food pantry as a coping mechanism [79,80]. However, some of these coping behaviors are not as common for young adults, even when considered to have very low food security [15]. Other researchers have attempted to assess ‘definitive classifications’ of FI to develop criterion validity [81,82], but this is labor-intensive and often requires subjective assessments from observers. In contrast, the current study compares how differing assessment protocols estimate FI prevalence and which protocol is best predicted by student characteristics that are theorized. This method of using determinants and consequences of FI has been conducted in other populations but provides the strongest indication of FI protocol accuracy when interpreted as part of a larger body of literature (as was done for the larger U.S. population in previous work) [58].

Considerations of FI assessment accuracy are important not only for researchers in the field, but also for universities interested in supporting increasingly diverse student populations. Many universities are enacting policies and interventions to address FI among their students [83]. Given the limited resources, both in terms of time and finances, available to many universities, it is vital that students with the greatest need are accurately identified. It is important that universities carefully consider which questionnaire they use not only for screening students but also for evaluating the effectiveness of any programs (such as campus food pantries or free dining hall meals) in alleviating FI. Though the 6- and 10-item USDA FSSMs have been commonly used in the past because they take little time to complete, minimize participant burden, and are simple to score, the current results suggest that including a 2-item screener may be valuable for assessing FI among post-secondary students in the field.

This research should be interpreted with considerations to the limitations of the study design and methodological choices. First, the online survey received a limited response rate. The study sample is comprised of those who decided to participate, which introduces selection bias. However, the sociodemographic characteristics of the sample had several similarities to the larger undergraduate student body. The largest difference between the sample and sampling pool was the greater proportion of female student participants, but women often respond at greater rates to surveys [84,85]. If questionnaires were administered in-person, a larger number of responses may have been collected. However, this methodology is much more time-intensive and still can pose its own issues with ascertaining a truly random sample. For example, prior FI assessment studies in college settings have recruited participants by selecting a list of courses [41,42], but this assumes all enrollees are in attendance. Ultimately, the recruitment efforts for this study resulted in a sample size that met power analysis needs. Aside from response rates, the study design attempted to identify an accurate measure of food security, but this is a subjective experience and thus is limited based on how accuracy is evaluated. Evaluations of these protocols in tandem with qualitative interviews and triangulation with other student risk factors and coping behaviors would provide further evidence for accuracy of assessment protocols. Further, generalizability of current results is limited by the single midwestern University sample. Therefore, this psychometric evaluation can, and should, be re-evaluated utilizing samples that include students from universities in various geographical and socio-economic settings. The limited population included in the current sample inhibits generalizability, but the homogeneity of the sample increases the internal validity of the results and overall statistical power to speak to traditional aged undergraduate students at a Midwest university in the U.S.

## Conclusions

Results from the current study indicate that the psychometric properties of FSSMs when used in college students were not ideal. These results warrant additional qualitative investigations of the FSSMs and possible adaptations for FI assessments in the college student population. Until these survey modifications are made, results of this protocol comparison analysis provide evidence that the way that FI is estimated among college students makes a substantial difference in reported prevalence, particularly with use of screeners. Given the limited resources available for universities to identify and serve students living with FI, it is vital that the way FI is assessed will accurately identify students most critically in need, such that the true impact of interventions may be evaluated. Current results suggest that the 10-item FSSM used along with the 2-item screener is the best currently available measure; however, replicating these analyses with a larger and more diverse sample is warranted.

## Supporting information

S1 Dataset Deidentified data for college food insecurity. (DTA)

S1 File Codebook for deidentified data for college food insecurity. (PDF)

S2 File Statistical protocol for all non-Rasch analyses in STATA 14.1 for college food insecurity. (DO)
