# Supplementary material for: Are estimates of food insecurity among college students accurate? Comparison of assessment protocols
Source: PLoS One. 2019 Apr 24;14(4):e0215161. doi: 10.1371/journal.pone.0215161 (PMC6481800; doi:10.1371/journal.pone.0215161)
Supplement: S1 File — (PDF) [file pone.0215161.s002.pdf]

```
. codebook, d

-------------------------------------------------------------------------------
ResponseId                                                           ResponseId
-------------------------------------------------------------------------------

                  type:  string (str17)

         unique values:  462                      missing "":  0/462

              examples:  "R_1lnINL9pLv8YjSR"
                         "R_2dEkygAybOIwMg8"
                         "R_3NWZauaWCgiBcgP"
                         "R_AEUp1I7jyQcc3CN"

-------------------------------------------------------------------------------
New_ID                                                                   New_ID
-------------------------------------------------------------------------------

                  type:  numeric (int)

                 range:  [101,562]                    units:  1
         unique values:  462                      missing .:  0/462

                  mean:     331.5
              std. dev:   133.512

           percentiles:        10%       25%       50%       75%       90%
                               147       216     331.5       447       516

-------------------------------------------------------------------------------
complete                                                  survey completed, yn
-------------------------------------------------------------------------------

                  type:  numeric (byte)
                 label:  yesno

                 range:  [0,1]                        units:  1
         unique values:  2                        missing .:  0/462

            tabulation:  Freq.   Numeric  Label
                            63         0  No
                           399         1  Yes

-------------------------------------------------------------------------------
classification                                                    college class
-------------------------------------------------------------------------------

                  type:  numeric (byte)
                 label:  class1

                 range:  [1,4]                        units:  1
         unique values:  4                        missing .:  0/462

            tabulation:  Freq.   Numeric  Label
                           126         1  Freshman
                           104         2  Sophomore
                           130         3  Junior
                           102         4  Senior

-------------------------------------------------------------------------------
fs_screen1_o                                          FI S1: run short of money
-------------------------------------------------------------------------------
```

```
                 type:  numeric (byte)
                label:  yesno

                range:  [0,1]                        units:  1
        unique values:  2                        missing .:  0/462

          tabulation:  Freq.   Numeric  Label
                         259         0  No
                         203         1  Yes
```

---

fs_screen2_o — **FI S2: food eaten in household**

```
                 type:  numeric (byte)
                label:  fsscreen2

                range:  [0,3]                        units:  1
        unique values:  4                        missing .:  0/462

          tabulation:  Freq.   Numeric  Label
                         232         0  Enough
                         203         1  Enough but not always
                          21         2  Sometimes
                           6         3  Often
```

---

fs4_o — **AD1: cut or skip meals**

```
                 type:  numeric (byte)
                label:  yesno

                range:  [0,1]                        units:  1
        unique values:  2                        missing .:  13/462

          tabulation:  Freq.   Numeric  Label
                         311         0  No
                         138         1  Yes
                          13         .
```

---

fs5_o — **AD1a: # days cut or skip meals**

```
                 type:  numeric (byte)

                range:  [1,25]                       units:  1
        unique values:  16                       missing .:  324/462

                 mean:   5.17391
             std. dev:   3.69634

          percentiles:        10%       25%       50%       75%       90%
                                2         3       4.5         6        10
```

---

fs6_o — **AD2: eat less than should**

```
                 type:  numeric (byte)
                label:  yesnodk
```

range: [0,2] units: 1
unique values: 3 missing .: 2/462

tabulation:

Freq.	Numeric	Label
327	0	No
117	1	Yes
16	2	Don't know
2	.	

---

fs7_o **AD3: hunger**

---

type: numeric (**byte**)
label: **yesnodk**

range: [0,2] units: 1
unique values: 3 missing .: 2/462

tabulation:

Freq.	Numeric	Label
362	0	No
86	1	Yes
12	2	Don't know
2	.	

---

fs8_o **AD4: lost weight**

---

type: numeric (**byte**)
label: **yesnodk**

range: [0,2] units: 1
unique values: 3 missing .: 3/462

tabulation:

Freq.	Numeric	Label
371	0	No
34	1	Yes
54	2	Don't know
3	.	

---

fs9_o **AD5: not eat whole day**

---

type: numeric (**byte**)
label: **yesnodk**

range: [0,2] units: 1
unique values: 3 missing .: 3/462

tabulation:

Freq.	Numeric	Label
432	0	No
21	1	Yes
6	2	Don't know
3	.	

---

fs10_o **AD5a: # days not eat**

---

type: numeric (**byte**)

```
        range:  [1,12]                    units:  1
unique values:  8                     missing .:  441/462

   tabulation:  Freq.  Value
                    6  1
                    3  2
                    3  3
                    1  4
                    5  5
                    1  9
                    1  10
                    1  12
                  441  .
```

---

d_gender_o **gender identity**

```
         type:  numeric (byte)
        label:  gender

        range:  [0,2]                     units:  1
unique values:  3                     missing .:  7/462

   tabulation:  Freq.   Numeric  Label
                  162         0  Male
                  290         1  Female
                    3         2  Other
                    7         .
```

---

d_gender_o_txt **string for 'other' gender**

```
         type:  string (str20)

unique values:  3                     missing "":  459/462

   tabulation:  Freq.  Value
                  459  ""
                    1  "Nonbinary"
                    1  "Nonbinary / demigirl"
                    1  "Transgender "

      warning:  variable has embedded and trailing blanks
```

---

birthcountry **Country of birth**

```
         type:  numeric (int)
        label:  born

        range:  [305,352]                 units:  1
unique values:  19                    missing .:  7/462

     examples:  305   America
                305   America
                305   America
                305   America
```

---

americatime **Time in U.S., if not born elsewhere**

```
              type:  numeric (byte)
             label:  time

             range:  [0,4]                        units:  1
     unique values:  5                        missing .:  386/462

        tabulation:  Freq.   Numeric  Label
                         6         0  Less than 1 year
                         6         1  1 to less than 2 years
                        17         2  2 to less than 5 years
                         8         3  5 to less than 10 years
                        39         4  10 years or more
                       386         .
```

---

d_residency_o **residence type**

```
              type:  numeric (byte)
             label:  reside

             range:  [1,5]                        units:  1
     unique values:  5                        missing .:  8/462

        tabulation:  Freq.   Numeric  Label
                        10         1  Home
                        22         2  Greek housing
                         5         3  Co-op
                       186         4  Dorm
                       231         5  Apartment
                         8         .
```

---

d_livesit_o **living situation**

```
              type:  numeric (byte)
             label:  livesit

             range:  [0,1]                        units:  1
     unique values:  2                        missing .:  8/462

        tabulation:  Freq.   Numeric  Label
                        47         0  Live alone
                       407         1  Live with other
                         8         .
```

---

mealplan **type of meal plan**

```
              type:  numeric (byte)
             label:  mealtype

             range:  [0,5]                        units:  1
     unique values:  6                        missing .:  9/462
```

```
      tabulation:  Freq.   Numeric  Label
                     269         0  No meal plan
                       7         1  6 meals per week
                      92         2  12 meals/15 credits per week
                      76         3  10 meals/45 credits per week
                       3         4  All credtis
                       6         5  Maximum meals
                       9         .
```

---

rateofhealth **self-rated health, 0-4**

```
            type:  numeric (byte)
           label:  rateheal

           range:  [0,4]                        units:  1
   unique values:  5                        missing .:  8/462

      tabulation:  Freq.   Numeric  Label
                       6         0  Poor
                      67         1  Fair
                     201         2  Average
                     154         3  Very good
                      26         4  Excellent
                       8         .
```

---

rateofdiet **self-rated diet, 0-4**

```
            type:  numeric (byte)
           label:  ratediet

           range:  [0,4]                        units:  1
   unique values:  5                        missing .:  8/462

      tabulation:  Freq.   Numeric  Label
                      37         0  Poor
                     111         1  Fair
                     205         2  Average
                      88         3  Very good
                      13         4  Excellent
                       8         .
```

---

freelunch **free/reduced lunches in high school**

```
            type:  numeric (byte)
           label:  yesno

           range:  [0,1]                        units:  1
   unique values:  2                        missing .:  17/462

      tabulation:  Freq.   Numeric  Label
                     359         0  No
                      86         1  Yes
                      17         .
```

SNAP **receive SNAP benefits**

```
                 type:  numeric (byte)
                label:  yesno

                range:  [0,1]                        units:  1
        unique values:  2                        missing .:  16/462

           tabulation:  Freq.   Numeric  Label
                          423         0  No
                           23         1  Yes
                           16         .
```

---

ttlincome **total parents annual income**

```
                 type:  numeric (byte)
                label:  income

                range:  [0,6]                        units:  1
        unique values:  7                        missing .:  96/462

           tabulation:  Freq.   Numeric  Label
                           12         0  Under $15000
                           38         1  $15000 to $34999
                           44         2  $35000 to $54999
                           56         3  $55000 to $74999
                           53         4  $75000 to $99999
                           80         5  $100000 to $149999
                           83         6  $150000 or more
                           96         .
```

---

firstgen **first-gen student**

```
                 type:  numeric (byte)
                label:  yesno

                range:  [0,1]                        units:  1
        unique values:  2                        missing .:  22/462

           tabulation:  Freq.   Numeric  Label
                          333         0  No
                          107         1  Yes
                           22         .
```

---

fin_other_txt **other financial sources listed**

```
                 type:  string (str37), but longest is str26

        unique values:  4                        missing "":  455/462

           tabulation:  Freq.  Value
                          455  ""
                            1  "Financial markets"
                            3  "Savings from previous work"
                            2  "Tuition Waiver"
                            1  "Varsity Athlete "

              warning:  variable has embedded and trailing blanks
```

fin_family_fq — **freq of support from family**

```
              type:  numeric (byte)
             label:  fin_freq

             range:  [0,3]                        units:  1
     unique values:  4                        missing .:  157/462

       tabulation:  Freq.   Numeric  Label
                       25         0  Per week
                       85         1  Per month
                       86         2  Per semester
                      109         3  Per year
                      157         .
```

fin_employ_fq — **freq of support from employment**

```
              type:  numeric (byte)
             label:  fin_freq

             range:  [0,3]                        units:  1
     unique values:  4                        missing .:  284/462

       tabulation:  Freq.   Numeric  Label
                       43         0  Per week
                       54         1  Per month
                       22         2  Per semester
                       59         3  Per year
                      284         .
```

fin_govt_fq — **freq of support from government**

```
              type:  numeric (byte)
             label:  fin_freq

             range:  [0,3]                        units:  1
     unique values:  4                        missing .:  341/462

       tabulation:  Freq.   Numeric  Label
                        2         0  Per week
                        3         1  Per month
                       58         2  Per semester
                       58         3  Per year
                      341         .
```

fin_schol_fq — **freq of support from scholarship**

```
              type:  numeric (byte)
             label:  fin_freq

             range:  [0,3]                        units:  1
     unique values:  4                        missing .:  306/462
```

```
      tabulation:  Freq.   Numeric  Label
                       2         0  Per week
                       2         1  Per month
                      57         2  Per semester
                      95         3  Per year
                     306         .
```

---
fin_loan_fq **freq of support from loans**

```
            type:  numeric (byte)
           label:  fin_freq

           range:  [0,3]                        units:  1
   unique values:  4                        missing .:  331/462

      tabulation:  Freq.   Numeric  Label
                       1         0  Per week
                       4         1  Per month
                      51         2  Per semester
                      75         3  Per year
                     331         .
```

---
fin_other_fq **freq of support from other**

```
            type:  numeric (byte)
           label:  fin_freq

           range:  [1,3]                        units:  1
   unique values:  3                        missing .:  459/462

      tabulation:  Freq.   Numeric  Label
                       1         1  Per month
                       1         2  Per semester
                       1         3  Per year
                     459         .
```

---
fin_other_ref **reference for amt/freq of 'other' support**

```
            type:  string (str27), but longest is str14

   unique values:  3                        missing "":  459/462

      tabulation:  Freq.  Value
                     459  ""
                       1  "Savings"
                       1  "Tuition Waiver"
                       1  "Tuition waiver"

         warning:  variable has embedded blanks
```

---
fin_family_amt **amount of support from family**

```
            type:  numeric (long)

           range:  [0,350000]                   units:  1
   unique values:  70                       missing .:  153/462
```

```
                  mean:     13705
             std. dev:   27317.1

          percentiles:        10%       25%       50%       75%       90%
                              100       400      5000     20000     35000

--------------------------------------------------------------------------
fin_employ_amt                           amount of support from employment
--------------------------------------------------------------------------

                  type:  numeric (long)

                 range:  [0,33000]                    units:  1
         unique values:  52                       missing .:  278/462

                  mean:   2035.14
             std. dev:    3733.12

          percentiles:        10%       25%       50%       75%       90%
                              100       200       500      2000      5000

--------------------------------------------------------------------------
fin_govt_amt                             amount of support from government
--------------------------------------------------------------------------

                  type:  numeric (double)

                 range:  [0,120000]                   units:  .1
         unique values:  45                       missing .:  338/462

                  mean:   7909.14
             std. dev:    11991.3

          percentiles:        10%       25%       50%       75%       90%
                             1000      2500      5000     10000     15000

--------------------------------------------------------------------------
fin_schol_amt                           amount of support from scholarship
--------------------------------------------------------------------------

                  type:  numeric (double)

                 range:  [0,40000]                    units:  .1
         unique values:  44                       missing .:  299/462

                  mean:    6711.5
             std. dev:    7370.07

          percentiles:        10%       25%       50%       75%       90%
                              500      1000      5000     10000     16000

--------------------------------------------------------------------------
fin_loan_amt                                  amount of support from loans
--------------------------------------------------------------------------

                  type:  numeric (long)

                 range:  [0,50000]                    units:  1
         unique values:  45                       missing .:  326/462

                  mean:   8688.87
             std. dev:    9289.16
```

```
    percentiles:        10%       25%       50%       75%       90%
                       1500      2500      5000     11500     22000
```

---

fin_other_amt — **amount of support from other**

```
           type:  numeric (int)

          range:  [100,16000]                  units:  100
  unique values:  3                        missing .:  459/462

     tabulation:  Freq.  Value
                      1  100
                      1  8000
                      1  16000
                    459  .
```

---

d_age_o — **Age, in years**

```
           type:  numeric (float)

          range:  [18,24]                      units:  1
  unique values:  7                        missing .:  0/462

     tabulation:  Freq.  Value
                    113  18
                    110  19
                    123  20
                     81  21
                     27  22
                      4  23
                      4  24
```

---

race_white — **Race, White**

```
           type:  numeric (float)
          label:  yesno

          range:  [0,1]                        units:  1
  unique values:  2                        missing .:  9/462

     tabulation:  Freq.   Numeric  Label
                    197         0  No
                    256         1  Yes
                      9         .
```

---

race_black — **Race, Black/African American**

```
           type:  numeric (float)
          label:  yesno

          range:  [0,1]                        units:  1
  unique values:  2                        missing .:  9/462
```

tabulation: Freq. Numeric Label

Freq.	Numeric	Label
425	0	No
28	1	Yes
9	.	

---

race_hisp — **Race, Hispanic or Latino/a**

---

type: numeric (**float**)
label: **yesno**

range: [**0**,**1**] units: **1**
unique values: **2** missing .: **9**/**462**

tabulation:

Freq.	Numeric	Label
396	0	No
57	1	Yes
9	.	

---

race_asian — **Race, Asian/Pacific Islander**

---

type: numeric (**float**)
label: **yesno**

range: [**0**,**1**] units: **1**
unique values: **2** missing .: **9**/**462**

tabulation:

Freq.	Numeric	Label
313	0	No
140	1	Yes
9	.	

---

race_other — **Race, Other**

---

type: numeric (**float**)
label: **yesno**

range: [**0**,**1**] units: **1**
unique values: **2** missing .: **9**/**462**

tabulation:

Freq.	Numeric	Label
451	0	No
2	1	Yes
9	.	

---

race — **Race, One Variable**

---

type: numeric (**float**)
label: **race1**

range: [**0**,**4**] units: **1**
unique values: **5** missing .: **9**/**462**

tabulation: Freq.   Numeric Label
233 **0** White
24 **1** Black/African American
44 **2** Hispanic or Latino/a
123 **3** Asian/Pacific Islander
29 **4** Other/Mixed
9 **.**

---

live_spouse **Spouse, YN**

---

type: numeric (**float**)
label: **yesno**

range: [**0**,**1**] units: **1**
unique values: **2** missing .: **60**/**462**

tabulation: Freq.   Numeric Label
395 **0** No
7 **1** Yes
60 **.**

---

live_roommate **Roommate(s), YN**

---

type: numeric (**float**)
label: **yesno**

range: [**0**,**1**] units: **1**
unique values: **2** missing .: **60**/**462**

tabulation: Freq.   Numeric Label
6 **0** No
396 **1** Yes
60 **.**

---

live_child **Child(ren), YN**

---

type: numeric (**float**)
label: **yesno**

range: [**0**,**1**] units: **1**
unique values: **2** missing .: **60**/**462**

tabulation: Freq.   Numeric Label
401 **0** No
1 **1** Yes
60 **.**

---

fs1_o **HH2: worry run out food**

---

type: numeric (**float**)
label: **fshh2**

range: [**0**,**2**] units: **1**
unique values: **3** missing .: **2**/**462**

```
      tabulation:  Freq.   Numeric  Label
                     311         0  Never True
                     129         1  Sometimes
                      20         2  Often
                       2         .
```

---

fs2_o — **HH3: food bought didnt last**

```
            type:  numeric (float)
           label:  fshh3

           range:  [0,2]                        units:  1
   unique values:  3                        missing .:  2/462

      tabulation:  Freq.   Numeric  Label
                     359         0  Never True
                      92         1  Sometimes
                       9         2  Often
                       2         .
```

---

fs3_o — **HH4: balanced meals**

```
            type:  numeric (float)
           label:  fshh4

           range:  [0,2]                        units:  1
   unique values:  3                        missing .:  10/462

      tabulation:  Freq.   Numeric  Label
                     293         0  Never True
                     113         1  Sometimes
                      46         2  Often
                      10         .
```

---

international — **RECODE of birthcountry (Country of birth)**

```
            type:  numeric (int)
           label:  yesno

           range:  [0,1]                        units:  1
   unique values:  2                        missing .:  7/462

      tabulation:  Freq.   Numeric  Label
                     378         0  No
                      77         1  Yes
                       7         .
```

---

socialclass — **family social class**

```
            type:  numeric (float)
           label:  soccls

           range:  [1,3]                        units:  1
   unique values:  3                        missing .:  14/462
```

```
          tabulation:  Freq.   Numeric  Label
                          42         1  Lower class
                         355         2  Middle class
                          51         3  Upper class
                          14         .
```

---

cc_xp **Experience with Community College**

```
                type:  numeric (float)
               label:  yesno

               range:  [0,1]                        units:  1
       unique values:  2                        missing .:  14/462

          tabulation:  Freq.   Numeric  Label
                         305         0  No
                         143         1  Yes
                          14         .
```

---

cc_hs **Community College in High School**

```
                type:  numeric (float)
               label:  yesno

               range:  [0,1]                        units:  1
       unique values:  2                        missing .:  14/462

          tabulation:  Freq.   Numeric  Label
                         383         0  No
                          65         1  Yes
                          14         .
```

---

cc_before **Community College before UIUC**

```
                type:  numeric (float)
               label:  yesno

               range:  [0,1]                        units:  1
       unique values:  2                        missing .:  14/462

          tabulation:  Freq.   Numeric  Label
                         406         0  No
                          42         1  Yes
                          14         .
```

---

cc_during **Community College during UIUC**

```
                type:  numeric (float)
               label:  yesno

               range:  [0,1]                        units:  1
       unique values:  2                        missing .:  14/462
```

tabulation:	Freq.	Numeric	Label
	**386**	**0**	No
	**62**	**1**	Yes
	**14**	**.**	

---

fin_family — **financial support from family**

---

type: numeric (**float**)
label: **yesno**

range: [**0**,**1**] units: **1**
unique values: **2** missing .: **13**/**462**

tabulation:	Freq.	Numeric	Label
	**67**	**0**	No
	**382**	**1**	Yes
	**13**	**.**	

---

fin_employ — **financial support from employment**

---

type: numeric (**float**)
label: **yesno**

range: [**0**,**1**] units: **1**
unique values: **2** missing .: **13**/**462**

tabulation:	Freq.	Numeric	Label
	**223**	**0**	No
	**226**	**1**	Yes
	**13**	**.**	

---

fin_govt — **financial support from government**

---

type: numeric (**float**)
label: **yesno**

range: [**0**,**1**] units: **1**
unique values: **2** missing .: **13**/**462**

tabulation:	Freq.	Numeric	Label
	**290**	**0**	No
	**159**	**1**	Yes
	**13**	**.**	

---

fin_schol — **financial support from scholarships**

---

type: numeric (**float**)
label: **yesno**

range: [**0**,**1**] units: **1**
unique values: **2** missing .: **13**/**462**

tabulation:	Freq.	Numeric	Label
	**236**	**0**	No
	**213**	**1**	Yes
	**13**	**.**	

```
--------------------------------------------------------------------------------
fin_loan                                          financial support from loans
--------------------------------------------------------------------------------

                  type:  numeric (float)
                 label:  yesno

                 range:  [0,1]                        units:  1
         unique values:  2                        missing .:  13/462

            tabulation:  Freq.   Numeric  Label
                           276         0  No
                           173         1  Yes
                            13         .

--------------------------------------------------------------------------------
fin_other                                 financial support from other sources
--------------------------------------------------------------------------------

                  type:  numeric (float)
                 label:  yesno

                 range:  [0,1]                        units:  1
         unique values:  2                        missing .:  13/462

            tabulation:  Freq.   Numeric  Label
                           441         0  No
                             8         1  Yes
                            13         .

--------------------------------------------------------------------------------
fin_family_sem                                support from family per semester
--------------------------------------------------------------------------------

                  type:  numeric (float)

                 range:  [0,175000]                   units:  .1
         unique values:  70                       missing .:  94/462

                  mean:   7921.06
             std. dev:   14433.5

           percentiles:        10%       25%       50%       75%       90%
                                 0       320      2500     11000     20000

--------------------------------------------------------------------------------
fin_employ_sem                            support from employment per semester
--------------------------------------------------------------------------------

                  type:  numeric (float)

                 range:  [0,16500]                    units:  1
         unique values:  52                       missing .:  61/462

                  mean:   893.835
             std. dev:   1599.87

           percentiles:        10%       25%       50%       75%       90%
                                 0         0         0      1500      2500

--------------------------------------------------------------------------------
fin_govt_sem                              support from government per semester
--------------------------------------------------------------------------------
```

```
                  type:  numeric (float)

                 range:  [0,160000]                   units:  .1
         unique values:  42                       missing .:  52/462

                  mean:   1979.74
             std. dev:    9004.79

          percentiles:        10%       25%       50%       75%       90%
                                0         0         0      1000   5434.75
```

---

fin_schol_sem — **support from scholarships per semester**

```
                  type:  numeric (float)

                 range:  [0,20000]                    units:  .1
         unique values:  42                       missing .:  72/462

                  mean:   1730.88
             std. dev:    3574.69

          percentiles:        10%       25%       50%       75%       90%
                                0         0         0      1500      6500
```

---

fin_loan_sem — **support from loans per semester**

```
                  type:  numeric (float)

                 range:  [0,60000]                    units:  1
         unique values:  44                       missing .:  56/462

                  mean:   1872.01
             std. dev:    4992.99

          percentiles:        10%       25%       50%       75%       90%
                                0         0         0      1750      5000
```

---

fin_other_sem — **support from other sources per semester**

```
                  type:  numeric (float)

                 range:  [0,8000]                     units:  100
         unique values:  3                        missing .:  18/462

           tabulation:  Freq.  Value
                          441  0
                            1  400
                            2  8000
                           18  .
```

---

finance_sem — **financial support per semester**

```
                  type:  numeric (float)

                 range:  [0,60000]                    units:  .1
         unique values:  190                      missing .:  18/462
```

mean: **10944.9**
std. dev: **10307.3**

percentiles:	10%	25%	50%	75%	90%
	**0**	**1000**	**9531.25**	**17000**	**23800**

---

fin_sup_ade **(unlabeled)**

---

type: numeric (**float**)
label: **foodins**

range: [**0**,**1**] units: **1**
unique values: **2** missing .: **0**/**462**

tabulation: Freq.	Numeric	Label
**302**	**0**	inadequate
**160**	**1**	adequate

---

bifs1_o **HH2: scored, binary**

---

type: numeric (**float**)
label: **yesno**

range: [**0**,**1**] units: **1**
unique values: **2** missing .: **2**/**462**

tabulation: Freq.	Numeric	Label
**311**	**0**	No
**149**	**1**	Yes
**2**	**.**	

---

bifs2_o **HH3: scored, binary**

---

type: numeric (**float**)
label: **yesno**

range: [**0**,**1**] units: **1**
unique values: **2** missing .: **2**/**462**

tabulation: Freq.	Numeric	Label
**359**	**0**	No
**101**	**1**	Yes
**2**	**.**	

---

bifs3_o **HH4: scored, binary**

---

type: numeric (**float**)
label: **yesno**

range: [**0**,**1**] units: **1**
unique values: **2** missing .: **10**/**462**

tabulation: Freq.	Numeric	Label
**293**	**0**	No
**159**	**1**	Yes
**10**	**.**	

bifs4_o — **AD1: scored, binary**

type: numeric (**float**)
label: **yesno**

range: [**0**,**1**] — units: **1**
unique values: **2** — missing .: **13**/**462**

tabulation:

Freq.	Numeric	Label
**311**	**0**	No
**138**	**1**	Yes
**13**	.	

bifs5_o — **AD1a: scored, binary**

type: numeric (**float**)
label: **yesno**

range: [**0**,**1**] — units: **1**
unique values: **2** — missing .: **13**/**462**

tabulation:

Freq.	Numeric	Label
**340**	**0**	No
**109**	**1**	Yes
**13**	.	

bifs6_o — **AD2: scored, binary**

type: numeric (**float**)
label: **yesno**

range: [**0**,**1**] — units: **1**
unique values: **2** — missing .: **2**/**462**

tabulation:

Freq.	Numeric	Label
**343**	**0**	No
**117**	**1**	Yes
**2**	.	

bifs7_o — **AD3: scored, binary**

type: numeric (**float**)
label: **yesno**

range: [**0**,**1**] — units: **1**
unique values: **2** — missing .: **2**/**462**

tabulation:

Freq.	Numeric	Label
**374**	**0**	No
**86**	**1**	Yes
**2**	.	

bifs8_o — **AD4: scored, binary**

```
           type:  numeric (float)
          label:  yesno

          range:  [0,1]                        units:  1
  unique values:  2                        missing .:  3/462

    tabulation:  Freq.   Numeric  Label
                   425         0  No
                    34         1  Yes
                     3         .
```

---

bifs9_o — **AD5a: scored, binary**

```
           type:  numeric (float)
          label:  yesno

          range:  [0,1]                        units:  1
  unique values:  2                        missing .:  3/462

    tabulation:  Freq.   Numeric  Label
                   438         0  No
                    21         1  Yes
                     3         .
```

---

bifs10_o — **AD5a: scored, binary**

```
           type:  numeric (float)
          label:  yesno

          range:  [0,1]                        units:  1
  unique values:  2                        missing .:  9/462

    tabulation:  Freq.   Numeric  Label
                   441         0  No
                    12         1  Yes
                     9         .
```

---

fs_sum_6 — **6-item FI sum**

```
           type:  numeric (float)

          range:  [0,6]                        units:  1
  unique values:  7                        missing .:  0/462

    tabulation:  Freq.  Value
                   238  0
                    63  1
                    35  2
                    25  3
                    37  4
                    30  5
                    34  6
```

---

fs_sum_10 — **10-item FI sum**

```
           type:  numeric (float)
```

```
           range:  [0,10]                       units:  1
   unique values:  11                       missing .:  0/462

            mean:   2.00433
       std. dev:     2.5873

     percentiles:        10%       25%       50%       75%       90%
                           0         0         1         4         6
```

---

fs_cat_6 — **6-item FI levels**

```
            type:  numeric (float)
           label:  fscats

           range:  [0,2]                        units:  1
   unique values:  3                        missing .:  0/462

      tabulation:  Freq.   Numeric  Label
                     301         0  High/Marginal
                      97         1  Low
                      64         2  Very Low
```

---

fs_cat_10 — **10-item FI levels**

```
            type:  numeric (float)
           label:  fscats

           range:  [0,2]                        units:  1
   unique values:  3                        missing .:  0/462

      tabulation:  Freq.   Numeric  Label
                     314         0  High/Marginal
                      80         1  Low
                      68         2  Very Low
```

---

fs_screenout — **if participant screens out**

```
            type:  numeric (float)
           label:  yesno

           range:  [0,1]                        units:  1
   unique values:  2                        missing .:  0/462

      tabulation:  Freq.   Numeric  Label
                     135         0  No
                     327         1  Yes
```

---

fs_cat_6sc — **6-item w/screener FI levels**

```
            type:  numeric (float)
           label:  fscats

           range:  [0,2]                        units:  1
   unique values:  3                        missing .:  0/462
```

```
     tabulation:  Freq.   Numeric  Label
                    370         0  High/Marginal
                     45         1  Low
                     47         2  Very Low
```

---

fs_cat_10sc — **10-item w/screener FI levels**

```
           type:  numeric (float)
          label:  fscats

          range:  [0,2]                        units:  1
  unique values:  3                        missing .:  0/462

     tabulation:  Freq.   Numeric  Label
                    374         0  High/Marginal
                     38         1  Low
                     50         2  Very Low
```

---

fs_cat_6scfin — **6-item w/screen&finance FI levels**

```
           type:  numeric (float)
          label:  fscats

          range:  [0,2]                        units:  1
  unique values:  3                        missing .:  0/462

     tabulation:  Freq.   Numeric  Label
                    328         0  High/Marginal
                     76         1  Low
                     58         2  Very Low
```

---

fs_cat_10scfin — **10-item w/screen&finance FI levels**

```
           type:  numeric (float)
          label:  fscats

          range:  [0,2]                        units:  1
  unique values:  3                        missing .:  0/462

     tabulation:  Freq.   Numeric  Label
                    335         0  High/Marginal
                     65         1  Low
                     62         2  Very Low
```

---

insec_6 — **Insecurity (0,1) from 6-item**

```
           type:  numeric (float)
          label:  insecurity

          range:  [0,1]                        units:  1
  unique values:  2                        missing .:  0/462

     tabulation:  Freq.   Numeric  Label
                    301         0  Secure
                    161         1  Insecure
```

insec_10 — **Insecurity (0,1) from 10-item**

```
                 type:  numeric (float)
                label:  insecurity

                range:  [0,1]                        units:  1
        unique values:  2                        missing .:  0/462

          tabulation:  Freq.   Numeric  Label
                          314         0  Secure
                          148         1  Insecure
```

insec_6sc — **Insecurity (0,1) from 6-item & screeners**

```
                 type:  numeric (float)
                label:  insecurity

                range:  [0,1]                        units:  1
        unique values:  2                        missing .:  0/462

          tabulation:  Freq.   Numeric  Label
                          370         0  Secure
                           92         1  Insecure
```

insec_10sc — **Insecurity (0,1) from 10-item & screeners**

```
                 type:  numeric (float)
                label:  insecurity

                range:  [0,1]                        units:  1
        unique values:  2                        missing .:  0/462

          tabulation:  Freq.   Numeric  Label
                          374         0  Secure
                           88         1  Insecure
```

insec_6scfin — **Insecurity (0,1) from 6-item & screeners & finances**

```
                 type:  numeric (float)
                label:  insecurity

                range:  [0,1]                        units:  1
        unique values:  2                        missing .:  0/462

          tabulation:  Freq.   Numeric  Label
                          328         0  Secure
                          134         1  Insecure
```

insec_10scfin — **Insecurity (0,1) from 10-item & screeners & finances**

```
                 type:  numeric (float)
                label:  insecurity
```

```
                 range:  [0,1]                        units:  1
         unique values:  2                        missing .:  0/462

            tabulation:  Freq.   Numeric  Label
                           335         0  Secure
                           127         1  Insecure

.
```
